# Behavioral and Autonomic Responses to Acute Restraint Stress Are Segregated within the Lateral Septal Area of Rats

**DOI:** 10.1371/journal.pone.0023171

**Published:** 2011-08-16

**Authors:** Daniel G. Reis, América A. Scopinho, Francisco S. Guimarães, Fernando M. A. Corrêa, Leonardo B. M. Resstel

**Affiliations:** Department of Pharmacology, School of Medicine of Ribeirão Preto, University of São Paulo, Ribeirão Preto, São Paulo, Brazil; Federal University of Rio de Janeiro, Brazil

## Abstract

**Background:**

The Lateral Septal Area (LSA) is involved with autonomic and behavior responses associated to stress. In rats, acute restraint (RS) is an unavoidable stress situation that causes autonomic (body temperature, mean arterial pressure (MAP) and heart rate (HR) increases) and behavioral (increased anxiety-like behavior) changes in rats. The LSA is one of several brain regions that have been involved in stress responses. The aim of the present study was to investigate if the neurotransmission blockade in the LSA would interfere in the autonomic and behavioral changes induced by RS.

**Methodology/Principal Findings:**

Male Wistar rats with bilateral cannulae aimed at the LSA, an intra-abdominal datalogger (for recording internal body temperature), and an implanted catheter into the femoral artery (for recording and cardiovascular parameters) were used. They received bilateral microinjections of the non-selective synapse blocker cobalt chloride (CoCl_2_, 1 mM/ 100 nL) or vehicle 10 min before RS session. The tail temperature was measured by an infrared thermal imager during the session. Twenty-four h after the RS session the rats were tested in the elevated plus maze (EPM).

**Conclusions/Significance:**

Inhibition of LSA neurotransmission reduced the MAP and HR increases observed during RS. However, no changes were observed in the decrease in skin temperature and increase in internal body temperature observed during this period. Also, LSA inhibition did not change the anxiogenic effect induced by RS observed 24 h later in the EPM. The present results suggest that LSA neurotransmission is involved in the cardiovascular but not the temperature and behavioral changes induced by restraint stress.

## Introduction

Stress situations are well-known to trigger autonomic and behavior responses that are accompanied by activation of several brain structures [1]. Among these structures, the lateral septal area (LSA) has been proposed as an integrative center for autonomic, neuroendocrine and behavioral responses [2], [3], [4]. The LSA projects into several brain regions involved in the modulation of autonomic and behavioral stress responses [4], [5], [6], [7], [8], [9]. The latter includes fear and anxiety responses and learning and memory interference [3], [4], [10], [11], [12], [13], [14], [15].

Acute restraint is an uncontrollable stress situation which produces several emotional and autonomic responses. The autonomic responses includes mean arterial pressure (MAP) and heart rate (HR) increases [7], [16], [17], [18], [19], [20], skeletal muscle vasodilatation and cutaneous vasoconstriction, which are accompanied by a rapid skin temperature drop and followed by body temperature increases [21], [22]. In addition to those autonomic responses, animals submitted to restraint also present behavioral changes such as reduced exploratory activity in an open field [23], [24], [25], increased immobility in a forced swimming test [26] and reduced exploration of the open arms of an elevated plus-maze (EPM) 24 h after the stress session [27], [28]. Therefore, it is possible evaluated the consequences of this stress model acutely by autonomic responses during restraint session and later, 24 h after the restraint session, by anxiogenic like effect in EPM.

Several studies have shown that the LSA is activated during aversive situations, including restraint stress [19], [29], [30], [31], [32]. Rats submitted to restraint stress exhibit increased c-Fos protein expression in LSA when compared with control animals [19]. In the same study, the authors reported that restraint stress caused an increase in blood pressure which was inhibited by the GABA A receptor agonist, muscimol, injected into the LSA. However, no changes on HR, body temperature and behavioral consequences evoked by restraint stress were described by LSA inhibition. Finally, anxiolytic-like effect induced by systemic administration of diazepam in rats submitted to fear conditioning model is associated with a decrease in LSA neuronal activity [29]. Taken together, these data support a possible regulatory role of the LSA on behavioral and autonomic responses associated with aversive situations such as the restraint stress.

Therefore, the aim of the present study was to investigate the involvement of the LSA in the autonomic and behavior responses induced by acute restraint stress.

## Materials and Methods

### Animals

Male Wistar rats weighing 230–250 g were used. The animals were kept in the animal care unit of the Department of Pharmacology, School of Medicine of Ribeirão Preto, University of São Paulo. The rats were housed individually in plastic cages with free access to food and water under a 12 h light/dark cycle (lights on at 06.30 h). The institution's Animal Ethics Committee approved the housing conditions and experimental protocols (protocol n. 150/2007).

### Surgery procedure

Seven days before the experiment, the rats were anesthetized with 2,2,2-tribromoethanol (Sigma, St Louis, Missouri, USA) (250 g/kg, intraperitoneally). After scalp anesthesia with 2% lidocaine, the skull was surgically exposed and stainless steel guide cannulae (0.55 mm) were implanted bilaterally into the LSA using a stereotaxic apparatus (Stoelting, Wood Dale, Illinois, USA) as described by [3]. During the surgical procedure an intra-abdominal datalogger (SubCue dataloggers, Calgary, Alberta, Canada) was also implanted to record internal body temperature. The rats were allowed to recover from the surgery during a period of seven days. Twenty-four h before the restraint stress (RS) session, the rats had a catheter (4 cm PE-10 segment heat-bound to a 13 cm PE-50 segment, Clay Adams, Parsippany, NJ, USA) inserted into the abdominal aorta through the femoral artery for blood pressure recording as described by Resstel et al. (2008). After each surgery, animals were treated with a polyantibiotic preparation of streptomycins and penicillins i.m. (Pentabiotico®, Fort Dodge, Brazil) to prevent infection and with the nonsteroidal anti-inflammatory flunixine meglumine (2.5 mg kg−1 s.c.; banamine®, Schering Plough, Brazil) for post-operative analgesia.

### Acute restraint

In the morning period (07:00–12:00 hr), the animals were transported to the experimental room in their home cages and allowed to adapt to this environment for at least 30 min. Mean arterial pressure (MAP) and heart rate (HR) were recorded with a HP-7754A amplifier (Hewlett Packard, Palo Alto, CA) connected to a signal acquisition board (Biopac M-100, Goleta, CA) for computer processing. After 15 min of baseline recording, rats received bilateral microinjection into the LSA of 100 nL of vehicle (sterile artificial cerebrospinal fluid (aCSF - composition: NaCl 100 mM; Na3PO4 2 mM; KCl 2.5 mM; MgCl2 1 mM; NaHCO3 27 mM; CaCl2 2.5 mM; pH = 7.4) or 1 mM/100 nL of CoCl_2_ (Sigma, St Louis, Missouri, USA) [3]. A 0.3 mm needle (Small Parts, Miami Lakes, Florida, USA), 1 mm longer than the guide cannula, connected to a 2 µL syringe (7001 KH; Hamilton Co., Nevada, USA) through a PE-10 tubing, was used for this purpose. The needles were carefully inserted into the guide cannulae and the solutions were infused over a 15 s period. They remained in place for an additional 30 s period to prevent reflux. Ten minutes later, the animals were submitted to a 60-min restraint period in a plastic cylindrical restraining tube (diameter 6.5 cm and length 15 cm). After restraint, the animals returned to their cages. Each animal was submitted to only one restraint session.

### Temperature measurements

Besides the cardiovascular parameters, variations in cutaneous temperature (CT) were recorded with the thermal camera Multi-Purpose Thermal Imager IRI 4010 (InfraRed Integrated Systems Ltd Park Circle, Tithe Barn Way Swan Valley Northampton, USA) at a distance of 50 cm. Internal temperature (IT) was recorded by the datalogger (SubCue dataloggers, Calgary, Alberta, Canada) implanted into the abdomen as described above.

### Elevated plus maze (EPM)

The EPM test was conducted as described before [28]. Briefly, the apparatus consisted of two opposite open arms (50×10 cm) crossed at a right angle by two arms of the same dimensions enclosed by 40 cm high walls with no roof. The maze was located 50 cm above the floor. Rodents naturally avoid the open arms of the EPM and anxiolytic compounds typically increase the exploration of these arms without changing the number of enclosed-arm entries [33], [34]. The AnyMazeTM software (version 4.7, Stoelting) was employed for behavioral analysis. It detects the position of the animal in the maze and calculates the number of entries and time spent in open and enclosed arms.

### Histological procedure

At the end of the experiments rats were anesthetized with urethane (1.25 g/kg, i.p.) and 100 nL of 1% Evan's blue dye was bilaterally injected into the LSA to stain the injection sites. The chest was surgically opened, the descending aorta occluded, the right atrium severed and the brain perfused with 10% formalin through the left ventricle. Brains were postfixed for 24 h at 4°C, and 40 µm sections were cut using a cryostat (CM-1900, Leica, Germany). Sections were stained with 1% neutral blue and injection sites were identified.

### Data Analysis

All autonomic responses were continuously recorded for 15 min before and during the 60-min of restraint stress period. Data were expressed as means ± SEM changes (respectively MAP, HR, CT or IT) and were sampled at 2 min intervals as a mean of the changes during each 2 min. Points sampled during the 10 min before restraint were used as control baseline value. The autonomic values changes during restraint were analyzed using two-way ANOVA with treatment as independent factor and time as repeated measurement factor. The basal values changes were analyzed before and after vehicle or CoCl_2_ administration by Student's t Test.

The percentage of entries (100×open/total entries) and time spent in the open arms (100×open/open+enclosed) of the EPM were calculated for each rat. These data, together with the number of enclosed arm entries, were analyzed by one-way ANOVA followed by Bonferroni's post hoc test. Values of P<0.05 were taken as showing statistically significant differences between means.

## Results

**Figure 1:**
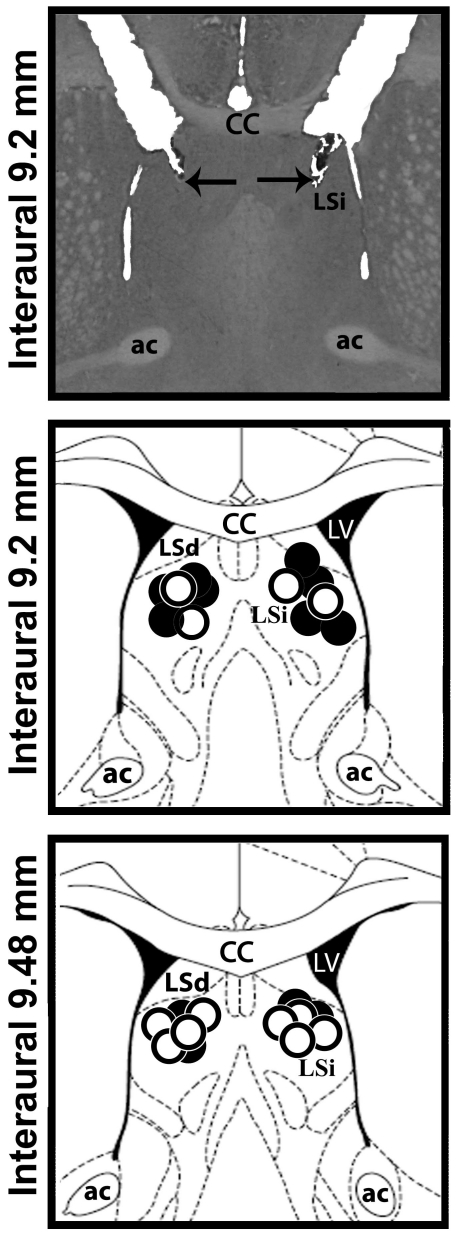
Anatomical sites of lateral septal area microinjections. A photomicrograph of a coronal brain section showing bilateral microinjections sites in the lateral septal area (LSA) and a diagrammatic representation based on the rat brain atlas of Paxinos and Watson (1997) indicating injections sites of vehicle (open circle) or CoCl_2_ (closed circle) into the LSA. cc- corpus callosum; LV- lateral ventricle; ac – anterior commissure; LSi - lateral septal area, intermediate part; LSd - lateral septal area, dorsal part.

A representative photomicrograph and a diagrammatic representation indicating the injection sites in the LSA can be seen in Figure 1.

### Effects of LSA inhibition on autonomic responses to acute restraint

**Figure 2:**
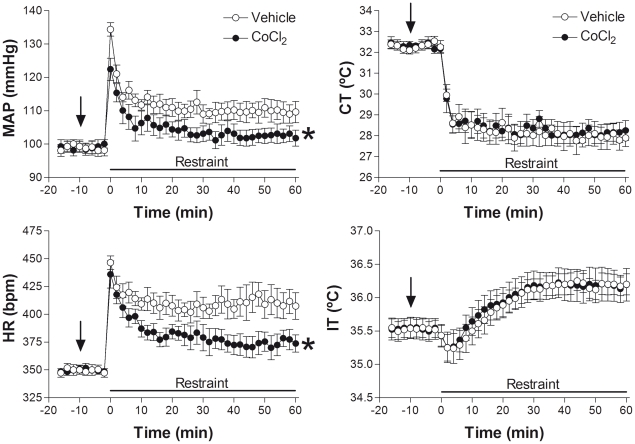
Autonomic responses during restraint stress. Time-course of bilateral microinjection of 100 nL of vehicle (n = 6) or 1 mM of CoCl_2_ (n = 6) on changes in mean arterial pressure (MAP), heart rate (HR), cutaneous temperature (CT) and internal temperature (IT) of animals submitted to 60 min of restraint stress. Symbols represent the means and bars the SEM. * P<0.05 Two-way ANOVA. The arrow represents the time of vehicle or CoCl_2_ administration.

**Figure 3:**
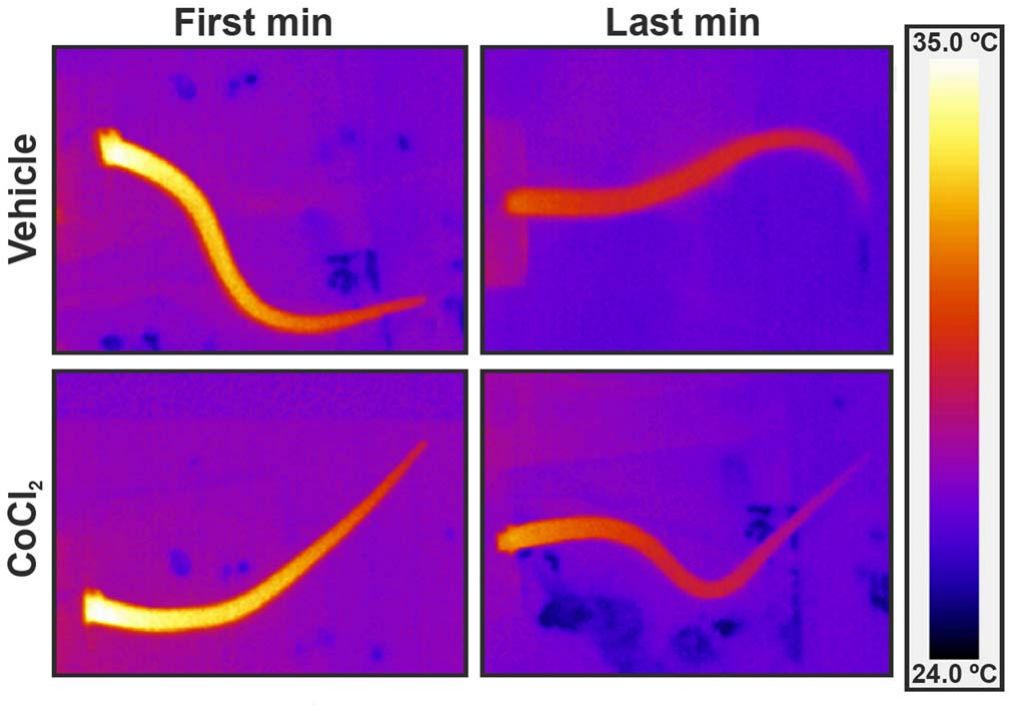
Infrared images of cutaneous temperature. Tail infrared digital images of representative rats which received either vehicle or CoCl_2_ into lateral septal area, during the first and last minute of restraint. Note the drop in cutaneous tail temperature during the restraint. All images use the same color coding for temperature.

The microinjection of CoCl_2_ into the LSA (n = 6) did not affect baseline values of either MAP (t = 0.32, P>0.05), HR (t = 1.85, P>0.05), CT (t = 0.45, P>0.05) or IT (t = 1.23, P>0.05). Acute restraint caused significant increases in MAP (F_35,360_ = 8.93, P<0.001), HR (F_35,360_ = 8.86, P<0.001) and IT (F_35,360_ = 5.86, P<0.001) and a significant and long-lasting decrease of CT (F_35,360_ = 20.11, P<0.001). The injection of CoCl_2_ into the LSA significantly attenuated the increases in both (MAP: F_1,360_ = 93, P<0.001) and (HR: F_1,360_ = 126, P<0.001, Figure 2) observed during restraint. However, no changes were observed in RS induced decrease of CT (F_1,360_ = 0.76, P>0.05) and increase of IT (F_1,360_ = 0.41, P>0.05, Figure 2). A representative infrared image of CT can be seen in Figure 3.

### Effects of LSA inhibition in the delayed anxiogenic effect in the EPM of restraint

**Figure 4:**
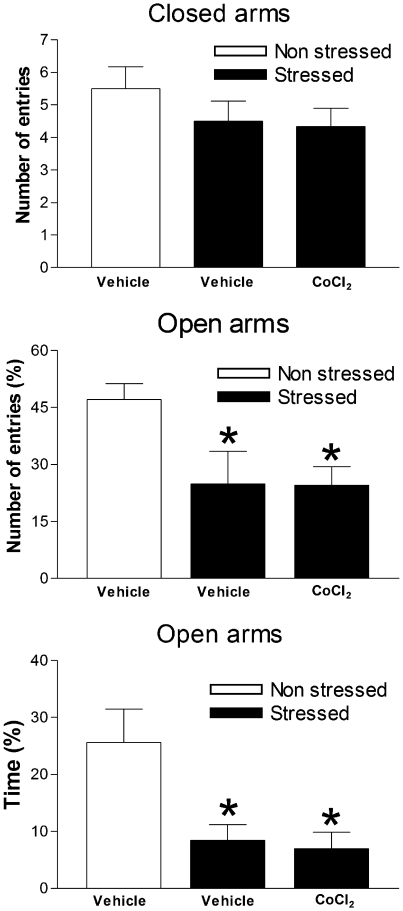
Consequences of restraint stress on behavioral responses. Effects of bilateral microinjection of 100 nL of vehicle (n = 6) or 1 mM of CoCl_2_ (n = 6) administered immediately before 60 min restraint period on behavior observed 24 h later in the elevated plus-maze (EPM). A non-stressed group was used as control. Columns represent the means and the bars the SEM. *P<0.05, Bonferroni's post-hoc test.

Animals submitted to acute restraint (n = 6) had a significant decrease in the percentage of time spent (F_2,17_ = 6.47, P<0.05) and in the number of entries in the open arms (F_2,17_ = 4.28, P<0.05) compared with unrestrained controls (n = 6). CoCl_2_ treatment (n = 6), however, failed to change these effects (P>0.05) Figure 4.

## Discussion

Solid and numerous evidences pointed that LSA is involved in the modulation of stress responses. The present work is the first study which analyzed several aspects of the autonomic and behavioral consequences associated by restraint stress. Our findings showing that LSA neurotransmission inhibition has a different consequence on autonomic responses observed during restraint stress, reducing hypertension and tachycardia without affecting both skin and body temperature changes. Moreover, LSA inhibition before restraint did not change the delayed increased anxiety behavior observed 24 h later in the EPM, suggesting that this inhibition failed to prevent behavioral consequences of stress exposure.

Our findings corroborate previous reports describing the autonomic and behavioral changes evoked by acute restraint [20], [35], [36]. In the present study the animals exhibited during the restraint period a significant increase in MAP and HR which were accompanied by a rapid CT drop followed by an increase in the IT. These changes reflect the activation of the autonomic nervous system that occurs during the exposure to an aversive situation [22], [37], [38].

Several studies demonstrated the activation of LSA during stress situations [19], [29]. The LSA can be subdivided in dorsal part, intermediate part and ventral part [12]. It is important to mention that in present study it was target the intermediate and dorsal parts of the LSA, avoiding reaching the ventral part due its proximity to the bed nucleus of stria terminalis [12], an area strongly involved with the cardiovascular modulation of restraint stress [18]. Despite that, it had shown that restraint stress stronger activates the medioventral part of the LSA compared to dorsal part [39]. Thus, the administration of CoCl_2_ in the ventral part of the lateral septal area could produce different effects on either autonomic responses or the stress-induced anxiogenesis. Therefore, we believe that more experiments are necessary to clarify the role of this LSA region, medioventral portion, on responses associated to restraint stress.

In the present study, we used CoCl_2_ to cause an acute and reversible LSA inhibition [3]. This compound has been employed to induce reversible inhibition of specific brain structures [9], [40], [41], [42]. CoCl_2_ causes a reversible inactivation that spreads over an area of about 0.1 to 2 mm^2^, by reducing Ca^2+^ pre-synaptic influx and thus interfering with neurotransmission release that leads to a synaptic blockage, without interference with fibers of passage [43], [44]. Thus, the use of CoCl_2_ to cause temporary inactivation of local neurotransmission can minimize several problems associated with lesion techniques, which could explain the differences between our results and previous reports.

It has been suggested that the LSA is an important central regulatory region for cardiovascular responses. Electrical stimulation of this region evokes blood pressure decreases or increases in anesthetized [45], [46] or unanesthetized rats [47], respectively. Cardiovascular changes are also reported after neurotransmitter agonist injections into the LSA, with glutamate decreasing [48] and acetylcholine, bradykinin and noradrenaline increasing MAP [49], [50], [51], [52], [53]. In addition, acute synaptic inhibition of the LSA enhances baroreflex responses, indicating an inhibitory influence of this region in this reflex [3].

Reinforcing the proposal that the LSA can regulate cardiovascular responses, the present results showed that acute neurotransmission inhibition in this region by local injection of CoCl_2_ reduces the cardiovascular changes observed during the restraint period. This result agrees with those from other studies reporting that LSA inactivation with muscimol reduces blood pressure increases during acute restraint stress [19] and during re-exposure to an aversive context [14]. Together they indicate that this region could be particularly related to the cardiovascular changes induced by stress exposure.

Contrasting with the decreased cardiovascular responses, LSA inhibition did not modify the stress-induced temperature changes. This finding indicates that during restraint stress the LSA could be modulating the activity of spinal sympathetic cardiomotor neurons but not that of sympathetic neurons controlling temperature changes. These latter neurons include those that control the cutaneous vascular tone in the tail skin and those that innervate the brown adipose tissue (BAT). Corroborating the present findings, distinct effects on sympathetic outflows to vasoconstrictor and thermoregulatory effectors have already been reported by several studies [54], [55], [56], [57], [58], [59]. Moreover, Beig et al. [37] found that systemic administration of a 5-HT_2A_ receptor antagonist prevents the stress-induced hyperthermia but does not change the MAP and HR increases in rats submitted to social defeat.

Acute restraint decreased the exploration of the open arms of the EPM 24 h after restraint stress without changing the number of enclosed arms. A decreased in the exploratory activity of new environments that follows a period of acute restraint stress has been described in several models, including the open field. It is sensitive to systemic and intra-cerebral injection of anxiolytic and antidepressant drugs, suggesting that it reflects a delayed anxiogenic effect [23], [24], [25], [27].

Distinct from the results observed with cardiovascular parameters, LSA inactivation did not change the anxiogenic effects of restraint stress. This finding was somehow surprising, since a wealth of evidence indicates that this region plays an important role in fear and anxiety [4], [11], [60], [61] and lesions of the lateral septum induce anxiolytic-like effects in the EPM and in the shock-probe test [10], [61]. In agreement with these results the LSA is densely interconnected with a number of limbic, diencephalic, and midbrain regions that regulate emotions and autonomic functions (Sheehan, Chambers et al. 2004, [62]. The LSA has reciprocal connections with the amygdala and receives projections from the hippocampus [4], [63], the medial prefrontal cortex [64] and the bed nucleus of the stria terminalis [15], areas involved in the autonomic changes observed during restraint stress [8], [18], [65]. The LSA is proposed to regulate motivated behaviors by integrating sensory stimuli and conveying this information to regions responsible for controlling and adjust these behaviors to environmental demands, for review see [4]. Our cardiovascular data clearly agree with this proposal. The results, however, also suggest that the delayed behavioral consequences of stressful stimulation depend on the engagement of other brain structures. Also, restraint or immobilization stress can induce intense expression of c-Fos in several brain areas such as medial prefrontal cortex, LSA, paraventricular and dorsomedial nuclei of the hypothalamus, retrochiasmatic area, medial and cortical nuclei of the amygdala, periaqueductal gray matter, and locus coeruleus (LC) [30], [66]. The intense activation of CNS has been related to complexity of the stress response. The activation of different brain structures can be part of facilitatory or inhibitory circuitry on the HPA axis [67], [68], [69], modulating differently the responses of stress. Future studies, comparing, for example, the changes in cFos activation during restraint stress induced by LSA inhibition, could be helpful to address this question.

Therefore, our findings indicate that during restraint stress LSA activity modulates the cardiovascular but not temperature responses. Moreover, this region also does not seem to be involved on the delayed anxiogenic consequences of this procedure.
